# Physiological Signal Monitoring Bed for Infants Based on Load-Cell Sensors

**DOI:** 10.3390/s16030409

**Published:** 2016-03-19

**Authors:** Won Kyu Lee, Heenam Yoon, Chungmin Han, Kwang Min Joo, Kwang Suk Park

**Affiliations:** 1 Interdisciplinary Program in Bioengineering, Graduate School, Seoul National University, Seoul 08826, Korea; wongyu86@snu.ac.kr (W.K.L.); hnyoon@bmsil.snu.ac.kr (H.Y.); cmhan616@gmail.com (C.H.); jkm6510@bmsil.snu.ac.kr (K.M.J.); 2 Department of Biomedical Engineering, College of Medicine, Seoul National University, Seoul 03080, Korea

**Keywords:** ballistocardiographs, physiological signal monitoring, infants, load-cell sensor, automatic optimal sensor selection algorithm

## Abstract

Ballistocardiographs (BCGs), which record the mechanical activity of the heart, have been a subject of interest for several years because of their advantages in providing unobtrusive physiological measurements. BCGs could also be useful for monitoring the biological signals of infants without the need for physical confinement. In this study, we describe a physiological signal monitoring bed based on load cells and assess an algorithm to extract the heart rate and breathing rate from the measured load-cell signals. Four infants participated in a total of 13 experiments. As a reference signal, electrocardiogram and respiration signals were simultaneously measured using a commercial device. The proposed automatic algorithm then selected the optimal sensor from which to estimate the heartbeat and respiration information. The results from the load-cell sensor signals were compared with those of the reference signals, and the heartbeat and respiration information were found to have average performance errors of 2.55% and 2.66%, respectively. The experimental results verify the positive feasibility of BCG-based measurements in infants.

## 1. Introduction

Healthcare technology continues to evolve toward the objective of constructing a routine health management system. An essential requirement of such a system is continuous physiological signal monitoring [1]. In the past, conventional methods of physiological measurement often caused discomfort by keeping subjects immovable or attaching various pieces of equipment to their bodies. However, these methods have been improved with the development of electronic technologies. Now, unobtrusive measurement technologies provide the possibility of managing subjects’ health status as they go about their day-to-day lives [2].

Researchers have been actively developing technologies to detect biological information from individuals without confining them or causing any discomfort. The most commonly studied methods for unobtrusive sensing include capacitive, photoplethysmographic, ballistocardiographic, and seismocardiographic approaches. These could become pervasive in our daily life without causing any discomfort through the use of wearable device (clothes and accessories) or commonly used objects (furniture and tools) [3,4]. These novel methods, which unobtrusively monitor the biological activity of subjects during their everyday life, can be used to set up a continuous examination system for long periods. They can also be used effectively for subjects who are difficult to confine physically, such as the elderly, infirm, and infants. In particular, it is expected that unobtrusive biomonitoring for infants could play an important role in managing breathing and cardiovascular problems, which are the main causes of death [5,6,7].

Conventional methods of physiological signal monitoring over long periods have limitations when applied to infants. For example, electrocardiography (ECG) using adhesive electrodes can cause skin allergies or inflammation. In some cases, the skin can be seriously damaged when detaching the electrodes from the body [6,8,9]. In addition, respiratory measurements can cause discomfort and irritation, as the equipment must be fastened firmly to the body [7]. Unobtrusive methods such as attaching a sensor to the nose [10] or foot (in the form of a sock) [11] have been used to measure biological information from infants. However, these methods still require the direct attachment of sensor devices to the body, which can be uncomfortable. Other indirect measuring methods include infrared thermography [7], piezoelectric force sensors [9], and Doppler radar [12]. Further investigations related to the clinical verification of these approaches is ongoing.

Ballistocardiographs (BCGs) represent a possible solution for monitoring infants’ physiological signals. BCGs are non-invasive instruments that measure the reactional motion of the body produced by cardiac contraction. Unlike ECGs, which require electrodes to be attached directly to the patient’s body to detect electric signals, BCGs generally use sensors for force, pressure, vibration, and displacement. These sensors do not require direct attachments, and therefore, they allow the measurement of physiological signals for long periods of time without perturbing the subject [4,13]. However, most studies investigating the application of these methods have focused on adults, and few cases have been focused on infants. More studies are necessary to verify the usefulness of BCG-based physiological measurement in infants.

Therefore, this study proposes a non-confining system for monitoring the biological information of infants using BCG technology. In a previous study, we developed a load-cell installed bed to measure the cardiac activity, respiration, and movement of adults [14,15]. The basic concepts of the present system, including the load-cell’s arrangement and the circuit design, are based on the former system. However, the detailed hardware specifications differ to account for relatively lightweight infant subjects. Because it is more difficult to obtain complete BCG signals from their immature hearts, which have smaller cardiac output than adults [16]. In addition, an algorithm that includes an automatic sensor selection with a signal quality check function has been developed to analyze the heart rate (HR) and breathing rate (BR) sagaciously from the four measured load-cell signals. The final objective is to validate and evaluate the feasibility of the infant biological information monitoring system. In experiments on infant subjects, the designed bed was used to acquire biological data, and the biological information from the proposed algorithm was compared with the reference information obtained simultaneously.

## 2. Materials and Methods

### 2.1. Proposed System

**Figure 1:**
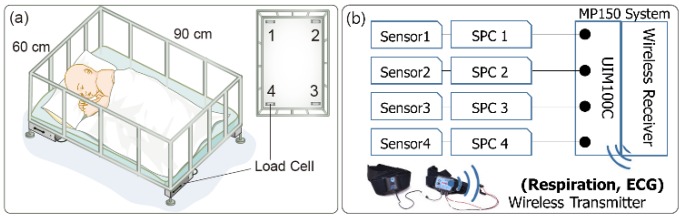
(**a**) Design of physiological signal monitoring bed for infants; (**b**) Experimental setup of signal acquisition system. (SPC: signal processing circuit)

Figure 1a illustrates the design of the physiological signal monitoring bed for infants proposed in this study. The size of the bed is 60 cm × 90 cm, and four load cells (CBCL-6L, Curiosity Technology, Paju-si, Gyeonggi-do, Korea) are installed below the plane of the bed. The load cells, which comprise four strain gauges in a Wheatstone bridge configuration, create an electrical signal in accordance with the force changes caused by cardiac activity and respiratory movements.

#### 2.1.1. Specifications of the Load-Cell Sensor

The technical specifications of the load-cell sensor are provided in Table 1. A single point load-cell, a type of resistive load-cell, is generally used in the industry, and it features superior off-center loading compensation. The gage factor is defined as the ratio of the fractional change in the electrical resistance to the given strain, and it is typically around 2 for metallic strain gages. Most manufacturers express the output of load-cell in units of mV/V, which is called the rated output. It is dependent on the gage factor and the operating stress of the load-cell structure, and it may range from 1 to 4 mV/V (a value of 2 mV/V is most common). For example, a load-cell with a rated output of 2 mV/V produces an output of 24 mV when supplied with excitation voltage of 12 V.

The sensitivity should be calculated based on the output and capacity of the load-cell as follows.

(1) $$Sensitivity\;of\;load-cell\;\left(mV/kg\right)=\frac{output}{capacity}=\frac{excitation\;voltage\;\left(V\right)\times rated\;output\;\left(mV/V\right)}{capacity\;\left(kg\right)}$$

In this study, each load-cell (CBCL-6L) with rated output of 2 mV/V and excitation voltage of 12 V shows sensitivity of 4 mV/kg.

#### 2.1.2. Signal Processing Circuit

**Figure 2:**
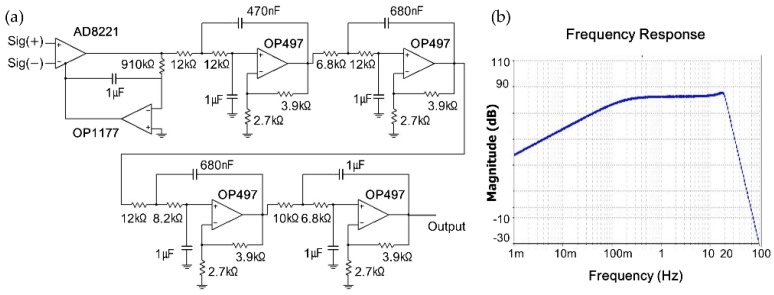
(**a**) Schematic diagram of signal processing circuit for the load-cell sensor; (**b**) Simulation results of frequency response of signal processing circuit.

Figure 2a illustrates the schematic diagram of the signal processing circuit for conditioning the load-cell output. Although the signals from load-cell sensors were amplified by the excitation voltage, the output caused by the cardiac activity and respiratory movements are not large enough to be displayed meaningfully. Thus, they were passed through an additional signal processing circuit. A precision instrumentation amplifier (AD8221, Analog Devices Inc., Norwood, MA, USA) with a gain setting of 100 was used in the circuit. This amplifier was associated with an alternating current (AC)-coupled circuit using an active integrator-based feedback network from the output to the reference terminal. This AC-coupled circuit improves the resolution of small AC signals by lowering the noise floor. Furthermore, OP1177, which forces the output of the AD8221 to 0 V at low frequencies, acts as a high-pass filter with a cut-off frequency of 0.1 Hz (please refer to the AD8221 datasheet). The signal output from the AD8221 was then passed through a low-pass filter to amplify the signal to the cardiorespiratory frequency range and suppress noise signals. This low-pass filter was composed of a Sallen–Key 8th-order Butterworth filter with a cut-off frequency of 20 Hz and a gain setting of 30. As a result, the overall circuit had a gain of 3000 and filtering bandwidth of 0.1–20 Hz. The simulation results of the frequency response of the signal processing circuit are shown in Figure 2b. Most of the signal acquisition methods described above follow the procedures described in the literature [17,18].

### 2.2. Subjects and Experimental Method

According to the Infant Care Act of Korea, preschool children under the age of six are considered infants. Four infants aged between 5 and 42 months (at the time of first participation) participated in the experiment after informed consent was received from their parents. Table 2 presents the physical characteristics of the four infants (labeled A through D) and experimental time. Because each subject participated more than once on different dates, a total of 13 experiments were conducted. The different dates for the same subject are indicated as, for example, B-1, B-2, and B-3. The experimental time shows somewhat irregular lengths. This was unavoidable because our experiments have some limitations related to the time constraints in accordance with the experimental conditions faced in home visits. Moreover, the subjects sometimes awoke easily when they were not in their own familiar bed. We have done our best to collect data for as long as possible.

During each experiment, the infant was laid down and allowed to fall asleep on the monitoring bed. As references to validate the performance of the developed bed and algorithm for analyzing the HR and BR, the signals from an electrocardiogram electrode and a breathing sensor (BN-RSPEC, BIOPAC Systems, Inc., Goleta, CA, USA) were recorded. ECG and breathing signals were transmitted wirelessly to the data acquisition system (MP150, BIOPAC Systems, Inc., Goleta, CA, USA) by using a receiver, and the four output signals of the developed system were also recorded simultaneously by the same data acquisition system (MP150 with UIM100C module). The MP150 system internally has a single ground connection and a microprocessor to control the data acquisition and communication with the computer. The wireless system has a separate ground for each transmitter. All data were collected at a sampling rate of 1000 Hz (Figure 1b).

**Figure 3:**
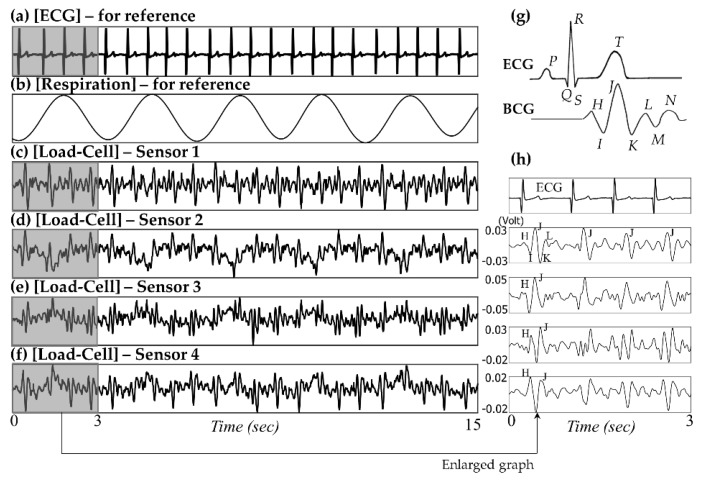
Simultaneous recordings of (**a**) ECG, (**b**) respiration signal, and (**d**)–(**f**) raw signals from four load-cell sensors, (**g**) typical BCG waveform (redrawn from literature [19]), and (**h**) waveform features of the BCG signal in our study.

Figure 3 shows a partial example of the monitored signals measured from a 19-month-old male subject, which is labeled as B-3. The output signals of the reference equipment (BN-RSPEC) are presented in (a)–(b), and the simultaneously acquired raw data from four load-cell sensors are presented in (c)–(f). Although the properties of the load-cell sensor outputs vary depending on a subject’s lying position, most sensors can record both ballistic cardiac activities (localized rapid fluctuations) and respiratory movements (slow oscillations in sensors 2 and 3). A detailed analysis is presented below in the discussion section. Figure 3g shows a typical BCG waveform from the literature and also shows some of the characteristic peaks in comparison with the ECG signal. Most named waveform features in the BCG signal are clearly shown in the magnified view of our example of monitored signals (Figure 3h).

### 2.3. Development of Algorithm for Automatically Analyzing HR and BR

**Figure 4:**
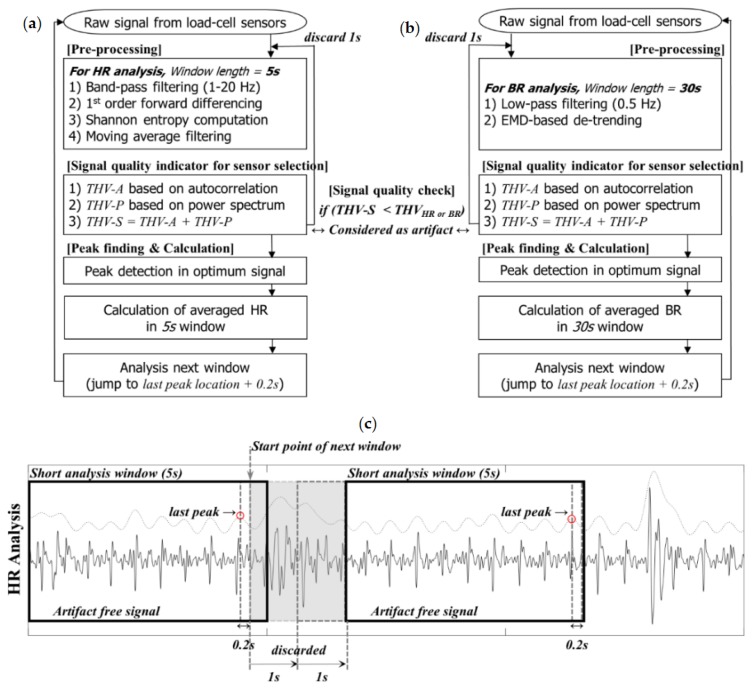
Flowchart of the proposed algorithms for automatic (**a**) HR and (**b**) BR analysis, (c) Partial example of HR analysis.

We designed an algorithm that performs automatic sensor selection with a signal quality check function to automatically analyze HR and BR. This algorithm consists of three stages: *pre-processing*, *signal quality check and sensor selection*, and *peak finding and calculation*. The basic concept of the proposed method is to iteratively shift a short analysis window across the signal. Although the overall flow applies equally to both heartbeat and respiration signal analysis, some details concerning the window length, pre-processing method, and criterion for artifact determination differ depending on the analysis objective. Figure 4 shows a flowchart outlining the algorithm used to estimate the HR and BR and its partial example captured when the HR analysis is being processed. The algorithm was independently operated for heartbeat and respiration signals (*i.e.*, there are two separate algorithms).

#### 2.3.1. Pre-Processing the Data

Consider the original signals obtained from the proposed system, $x_{k}\left[n\right]$ (k=1,⋯,4, the number of load-cell sensors), where n is the length of the analysis window. For the HR and BR analysis, we set n to 5 s and 30 s, respectively. Considering a sampling rate of 1000 Hz, 5000 samples were included in each HR analysis window, and 30,000 samples were included in each BR analysis window. In the general case, the signal $x_{k}\left[n\right]$ includes components of cardiac activity (usually called BCG) and respiratory movement. The pre-processing stage applies an infinite impulse response (IIR) digital filter to extract the appropriate physiological rhythm, and it employs other signal processing techniques to enhance the peak component and suppress noise. To avoid any time delay or phase shift, zero-phase digital filtering (offered by the “filtfilt” function in the MATLAB software) was applied in all the following filtering processes. Zero-phase digital filtering, which was conducted by processing the input data in both the forward and the reverse directions, reduces noise in the signal and preserves the peak at the same time at which it occurs in the original.

##### Pre-Processing for the HR Analysis

**Figure 5:**
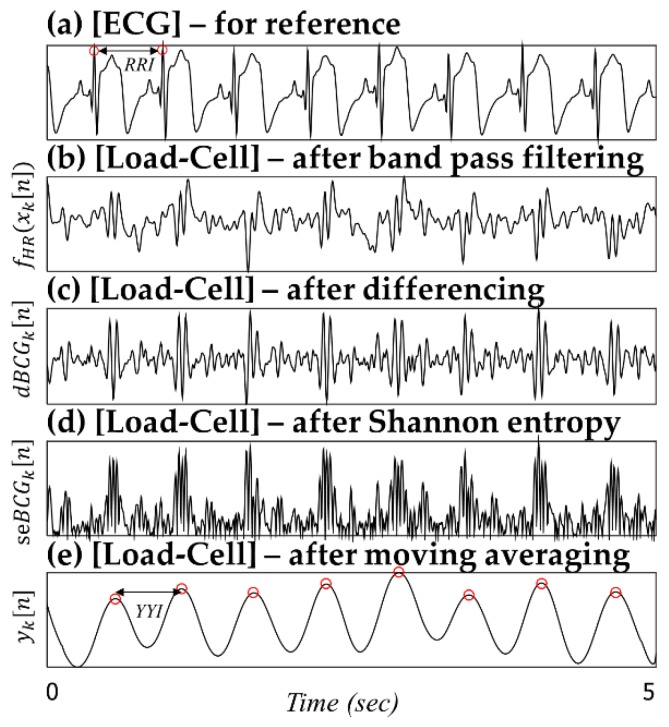
Pre-processing steps for the HR analysis.

The signal is first separated using a fifth-order band-pass Butterworth filter (IIR) with a range of 1–20 Hz to extract clear heartbeat derived signals (Figure 5b), denoted as $f_{HR}\left(x_{k}\left[n\right]\right)$. Subsequently, first-order differentiation is applied to the signals $f_{HR}\left(x_{k}\left[n\right]\right)$ to extract information about the slope and accentuate the main peak component (Figure 5c), denoted as $dBCG_{k}\left[n\right]$). The $dBCG_{k}\left[n\right]$ signals are implemented as

(2) $$dBCG_{k}\left[n\right]=f_{HR}\left(x_{k}\left[n+1\right]\right)-f_{HR}\left(x_{k}\left[n\right]\right)$$

The differentiated signal ($dBCG_{k}\left[n\right]$) is passed through a nonlinear transformation to obtain positive peaks regardless of the polarity of the main peak components (Figure 5d), denoted as $seBCG_{k}\left[n\right]$). The main objective of this transformation is to enhance the main peak components and use a single-sided threshold mechanism. The nonlinear transformation giving the Shannon entropy value is applied as follows:

(3) $$seBCG_{k}\left[n\right]=-\left|dBCG_{k}\left[n\right]\right|\times log\left(\left|dBCG_{k}\left[n\right]\right|\right)$$

Finally, a moving average filter is applied to smooth out the spikes and noise bursts (Figure 5e), denoted as $y_{k}\left[n\right]$). The signal quality check and sensor selection stage then determine the optimum signal, and the corresponding $y_{k}\left[n\right]$ signal is passed to the peak finding and calculation stages.

In Figure 5, for example, according to the seven successive RR intervals (intervals between the R-peaks from the ECG signal), the average RR interval (RRI) from the ECG signal is 0.5874 ± 0.0169 s. In contrast, the average YY interval (YYI) from the $y_{k}\left[n\right]$ signal is 0.5851 ± 0.0226 s. Additionally, the average difference between RRI and YYI (|RRI-YYI|) is 0.0103 ± 0.0069 s. Heartbeat information can be extracted quite well by applying the above data processing stage. This preconditioning is useful for handling BCG signals with less-dominant peak components caused by small heartbeat amplitudes.

##### Pre-Processing for the BR Analysis

**Figure 6:**
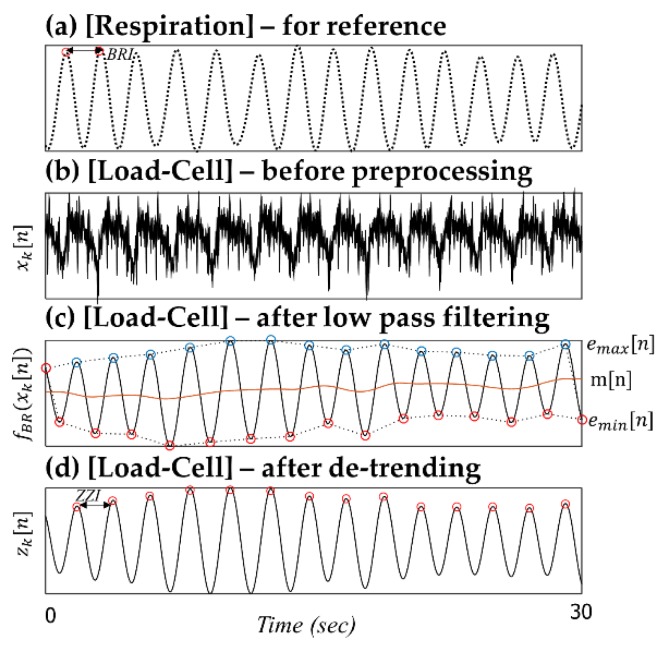
Pre-processing steps for BR analysis.

For the BR analysis, the raw signals $x_{k}\left[n\right]$ are first discerned using a fifth-order 0.5-Hz low-pass Butterworth filter (IIR) to extract the main respiratory rhythm (Figure 6, denoted as $f_{BR}\left(x_{k}\left[n\right]\right)$). Although the original signals obtained from the developed system ($x_{k}\left[n\right]$) have already passed through the electronic high-pass filter (AC-coupled circuit, fc = 0.1 Hz), some remaining low-frequency components can cause the baseline to wander. To suppress unwanted residual baseline drift, a de-trending technique based on empirical mode decomposition (EMD) is used to preprocess the signal $f_{BR}\left(x_{k}\left[n\right]\right)$ [20].

Hereafter, the de-trended $f_{BR}\left(x_{k}\left[n\right]\right)$ signal is denoted as $z_{k}\left[n\right]$. As in the HR analysis, the peak finding and calculation stage are conducted according to the $z_{k}\left[n\right]$ signal corresponding to the optimum signals selected by the signal quality check and sensor selection stages. In Figure 6, for example, according to the 13 successive breathing intervals (BRI), the average breathing interval from the respiratory signal is 2.0945 ± 0.1354 s. In contrast, the average ZZI from the $z_{k}\left[n\right]$ signal is 2.1015 ± 0.0945 s. The average difference between BRI and ZZI (|BRI-ZZI|) is 0.0545 ± 0.0349 s.

#### 2.3.2. Signal Quality Check and Sensor Selection

The most important aspect of the signal analysis obtained from unobtrusive sensors is the signal quality. In particular, the signals obtained from the developed load-cell-based system are much weaker than other types of body signals. Therefore, it is important to separate that portion of the signal that includes useful biological information from the artifact component.

If the obtained signal is clearly recorded without artifacts, it can be assumed to exhibit special periodicity according to the heartbeat or respiratory cycles. Thus, the main objective of this stage is to determine whether the signal contains periodicity in the expected frequency region. The expected frequency region is set differently for HR and BR analysis. Let $f_{l}$ and $f_{h}$ represent the lower and higher frequencies of the expected frequency region. For HR analysis, these parameters were set to 0.8 and 2 Hz, respectively, whereas for the BR analysis, they were set to 0.2 and 0.8 Hz, respectively.

**Figure 7:**
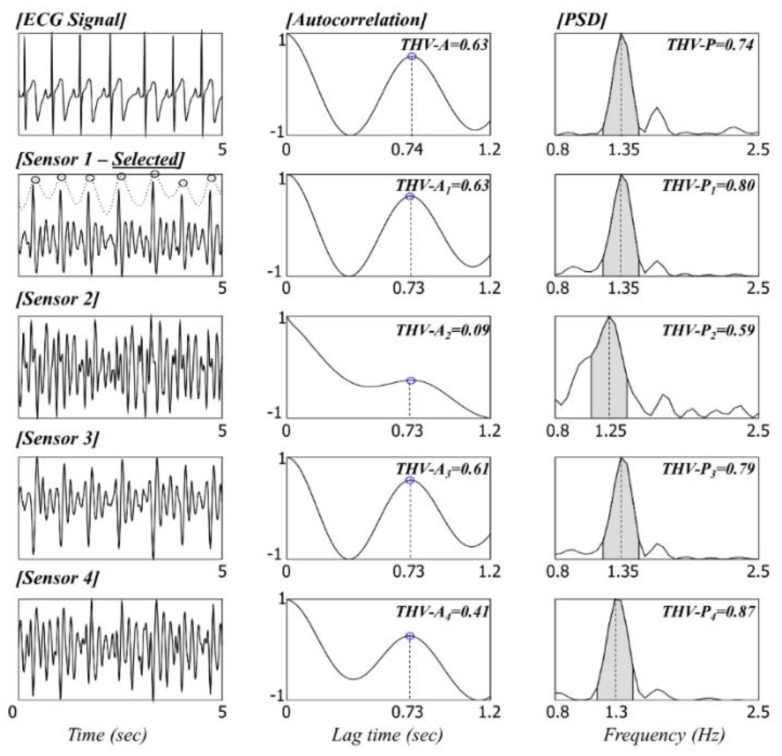
Signal quality check based on the THV-S and sensor selection for HR analysis.

**Figure 8:**
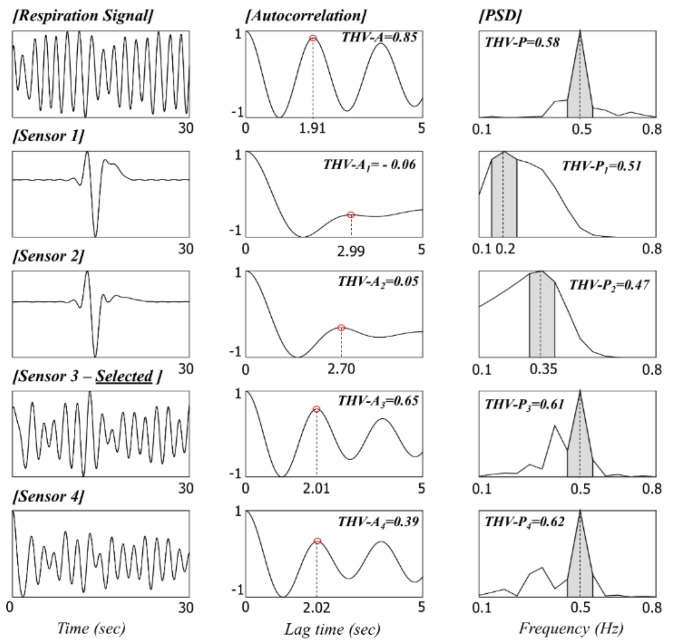
Signal quality check based on THV-S and sensor selection for BR analysis (There are motion artifacts in the signals from sensors 1 and 2, and they were eventually excluded in the following sensor selection by the lower THV-S).

In addition, we developed signal quality indicators (THV-S_k_) by using the calculated threshold parameters based on the autocorrelation (THV-A_k_) and power spectral densities (THV-P_k_). The indicators were used for estimating the level of artifacts in the following signal quality check and sensor selection processes. The following details of signal quality calculations for optimal sensor selection are illustrated in Figure 7 (HR) and Figure 8 (BR).

##### Autocorrelation-Based Threshold

The autocorrelation function (ACF) measures the correlation between $b_{t}$ and $b_{t+i}$, where i=0, ⋯, j. The autocorrelation formula ($r_{j}$) for lag j is

(4) $$r_{j}=c_{j}/c_{0}$$

where $c_{j}=\frac{1}{T-1}\overset{T-j}{\underset{t=1}{\sum }}\left(b_{t}-\bar{b}\right)\left(b_{t+j}-\bar{b}\right)$, $c_{0}$ is the sample variance of the time series, and T is the total number of samples in the window for the ACF calculation. The lag j is determined using the lower expected frequency region explained above. For the HR and BR analysis, j was set to 1.25 s (=0.8 Hz) and 5 s (=0.2 Hz), respectively.

If the signal is clean and contains the periodicity of the heartbeat and respiratory cycles, peaks appear in the ACF graph (when the first derivative of ACF goes from positive to negative). In the case of multiple peaks, only the first is considered to represent the periodicity. When the position of the peak in the ACF is in the predetermined range of 0.5–1.25 s lag for HR and 1.25–5 s lag for BR, the algorithm regards the position to be normal. The ACF value at that peak is recorded as THV-A_k_. If there is no peak in the range, THV-A_k_ is set to 0. Such signals are often determined as being artifacts in the following signal quality check stage.

##### Power Spectral Density (PSD)-Based Threshold

The PSDs of data within a short analysis window are calculated by a fast Fourier transform (FFT) method. We use a periodogram containing 20,000 discrete Fourier transform (DFT) points, which gives a spectral resolution of 0.05 Hz. The PSD-based threshold THV-P_k_ is defined as the concentration ratio of the power summation. First, the maximum PSD in the expected frequency region, between $f_{l}$ and $f_{h}$, is detected. If the frequency with the maximum power is denoted as $f_{max}$, then THV-P_k_ is computed as

(5) $${\text{THV-P}}_{k}=\sum_{f=f_{max}-0.1}^{f_{max}+0.1}P_{f}/\sum_{f=0.1}^{f_{h}}P_{f}$$

##### Signal Quality Check and Selection of Optimum Sensor Channel

The signal quality check is conducted based on THV-S_k_ (THV-S_k_ = THV-A_k_ + THV-P_k_). If THV-S_k_ is larger than the predetermined values of THV_HR_ and THV_BR_, the signal included in the current short window (of length 5 or 30 s for HR or BR, respectively) is considered to be clean. In this study, THV_HR_ and THV_BR_ were empirically set to 0.65 and 1.25, respectively.

If all THV-S_k_ values are lower than the predetermined threshold, the signal in the current short window is considered to be an artifact. The short analysis window then slides along for 1 s until a clean signal is detected (*i.e.*, start point of window changes from a_1_ to a_1_ + 1 s). If more than one THV-S_k_ value from the four load-cell sensors is higher than the predetermined threshold value, the sensor with the maximum THV-S_k_ value is selected as the most suitable sensor for measuring the cardiac activity or respiratory movement. Then, the peak included in the current short window of the most suitable sensor is detected, and the intervals between peaks are calculated. The short analysis window then jumps to the next start point. To prevent the case in which the peak may be located at the edge of the window, the next start point was set by considering the location of the last peak in the current window (for example, location of last peak + 0.2 s, as also shown in Figure 4c).

#### 2.3.3. Performance Evaluation

To evaluate the performance of the developed algorithm, the peak information from both the ECG and the respiratory signals (from the chest belt sensors) were labeled by researchers. An internally developed MATLAB-based software tool featuring automatic peak detection, manual peak marking, and correction was used in this peak annotation process. Information about the peak locations and derived intervals between peaks were used as a standard reference in the following performance evaluation.

First, the reliability of the proposed algorithm with respect to the beat locations was assessed using the following quantitative indexes. For the heartbeat location, we defined the beat location evaluation area that ranges from R peak location −0.15 s to R peak location +0.35 s. The beat location evaluation area considered the different peak timings derived from a dissimilar signal source, ECG (electrical signal), and BCG (mechanical signal). If the closest load-cell-derived heartbeat was within this beat location evaluation area, it was denoted as a true positive (TP), *i.e.*, it was correctly detected by the proposed algorithm. Other unassigned load-cell-derived heartbeats were counted as false positives (FPs). When no heartbeats were within the beat location evaluation area corresponding to an R peak in the ECG, it was counted as a false negative (FN). This process was similarly applied to the load-cell-derived respiratory signals. However, the beat location evaluation area for BR analysis was set to range from respiratory peak location −0.45 s to respiratory peak location +1.05 s.

**Figure 9:**
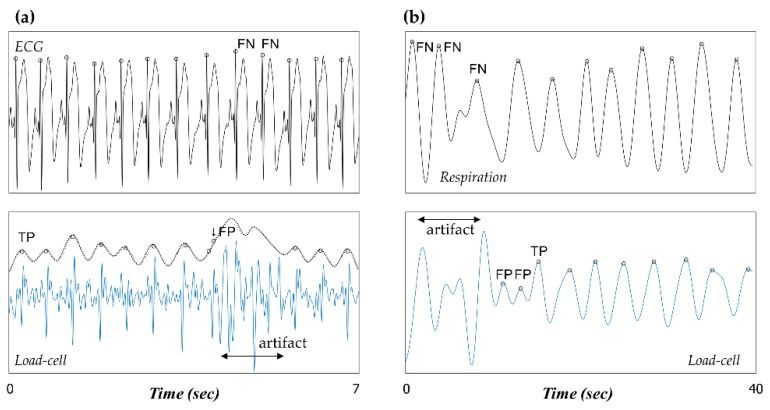
Manually annotated peak locations and automatic peak detection results of the load-cell sensor signal obtained by the developed algorithm in (**a**) HR analysis and (**b**) BR analysis (definitions of TP, FP, and FN are illustrated).

Figure 9 shows a comparison between the manually annotated peak locations and the automatic peak detection results of the load-cell sensor signal given by the developed algorithm. The detection rate (Dr) and positive predictive value (PPV) were calculated as follows:

(6) $$Dr=\;\frac{\;TP_{\left(from\;load-cell\;signal\right)}}{Total\;number\;of\;beat_{\left(ECG\;or\;Chest\;belt\right)}}\times 100\;\left(\%\right)$$

(7) $$PPV=\;\frac{TP}{TP+FP}\times 100\;\left(\%\right)$$

Second, the adequacy of the proposed algorithm with respect to regular HR or BR monitoring was evaluated. Our proposed algorithm uses a short analysis window of 5-s and 30-s lengths for HR and BR, respectively. For each non-overlapping 5-s (30-s) window, the difference and error between the mean HR (BR) derived from the load-cell sensor signal during that window and that derived from the ECG (respiratory signal from chest belt sensor) was computed. These differences and errors were then aggregated during each experiment. Finally, for each experiment, the mean absolute difference and mean absolute error were reported.

The coverage denotes the percentage of the signal that is automatically classified as clean. As mentioned above, to obtain reliable HR and BR signals from load-cell sensors, periods with poor signal quality are discarded during the signal quality check and optimum sensor selection step. The coverage was calculated as follows:

(8) $$coverage\;(\%)=\;\frac{periods\;with\;clean\;signal\;\left(s\right)}{Total\;periods\;of\;the\;experiment\;\left(s\right)}=\;\frac{Total\;periods\;of\;experiment\;\left(s\right)-periods\;with\;artifact\;signal\;\left(s\right)}{Total\;periods\;of\;the\;experiment\;\left(s\right)}$$

## 3. Results

We analyzed 13 records from four infant participants. The signals collected from the load-cell sensors include the components caused by cardiac activity and respiratory movement. To analyze the biological information, we also developed a MATLAB-based automatic algorithm that has been validated experimentally.

Table 3 and Table 4 show the results of the developed algorithm with respect to the beat locations for each experiment (see Section 2.3.3). The algorithm achieved an average detection rate of 76.16% (Table 3, HR) and 89.35% (Table 4, BR) and an average positive predictive value of 99.07% (Table 3, HR) and 96.22% (Table 4, BR).

As in the case of many unobtrusive monitoring, the signals acquired by our load-cell system are prone to high-grade noise. The main artifacts are related to movement, which is an inevitable consequence of physiological monitoring. Thus, the most important aspect of signal analysis using unobtrusive sensors is to classify the good-quality signals. In our results, FN is defined as the failure to detect an existing ECG or respiratory peak. It is assumed that most FNs were the result of our algorithm automatically determining an analysis window to be a noise signal and discarding all peaks included in the window. We additionally investigate how FN results inevitably occur in the artifact area and are denoted as FN (inevitable). 

However, the number of FPs and FNs gives a limited indication of the accuracy of the estimated HR and BR. We estimate that FN and FP directly affect the lengths of their neighboring beat-to-beat intervals. Therefore, the overall quality of the estimated HR and BR monitoring was assessed by the mean difference and error in computing the intervals extracted from the load-cell sensors and the corresponding intervals given by the reference equipment. 

Table 5 and Table 6 show the adequacy of the proposed algorithm with respect to regular HR or BR monitoring. On average, 73.79% and 84.25% of each signal was identified as being artifact-free and usable for HR or BR estimation, respectively. The results indicate that the mean errors of the estimated HR averaged over 5 s and the estimated BR averaged over 30 s were 2.55% and 2.66%, respectively.

Some other related results cited in the literature are summarized in Table 7. However, it is impossible to make an objective comparison between our results and those in Table 7, which were obtained under different experimental circumstances. In particular, most of the results (Records 02–07) were obtained from adult subjects. The differences in subject details, recording times, and sensors used in the study are represented in this table.

## 4. Discussion and Conclusions

After the basic concept of BCG was introduced in 1877, unobtrusive physiological measurement using BCG technology has become an interesting topic that has attracted much research attention. Recently, a review of numerous studies on BCG has been presented in [4]. However, it is rare to find studies that have focused on monitoring the biological signals of infants. As mentioned in the introduction section, BCG technology could play a more important role when applied to infants who are difficult to confine physically. We believe that the proposed device and algorithm presented herein are essential for realizing the unobtrusive monitoring of infants.

In this study, we developed a load-cell installed bed to measure the cardiac activity, respiration, and movement of infants, as illustrated in Figure 1. The basic concepts of the present system, including the load-cell’s arrangement and the circuit design, are based on our former system [14,15]. However, the detailed hardware specifications differ to account for the relatively low weight of the infant subjects. At the beginning of our study, we attempt to revamp an existing monitoring system. This system was built with four load-cell sensors (MNC-100L, CAS, Yangju-si, Gyeonggi-do, Korea) installed under the legs of the bed. The capacity of the load-cell was 100 kg, and its rated output is 2 mV/V. Although it was well suited for detecting the subtle body vibrations of adults, we could not discern any useful signal for applying the same system to infants. We suppose that the high capacity of the load-cell for covering the entire weight, including that of the bed, leads to lower sensitivity, which is not appropriate for measuring the subtle body vibrations of infants. Considering the few characteristics that affect the load-cell sensitivity (see Section 2.1), it is relatively easy to reduce the capacity of the load-cell. We designed a simple load-cell system (capacity of 6 kg) while retaining the essential functions, thus we expect a theoretical system capacity of only 24 kg for realizing an improvement in the sensitivity. In the commercialization stage, we plan to incorporate our developed device into the body of a commercial infant’s bed.

We could finally measure cardiac and respiratory signals by using load-cell sensors. Figure 3 shows a partial example of the monitored signals measured from a 19-month-old male subject. According to the observational record, the subject lay down in bed in a somewhat diagonal direction. His head was located near sensor 1, the region of the right shoulder and heart was located near sensor 2, the region of the hip and right leg was located near sensor 3, and the left leg was located near the middle of sensors 3 and 4 (please refer to the sensor locations in Figure 1a). Because the chest and stomach are biased to the region of sensors 2 and 3, the respiratory signals are observed as relatively distinct shapes in the corresponding channels. Furthermore, there are phase differences in the recorded respiratory signals between sensors 2 and 3. As already discussed in several literatures, the respiration signals could have phase differences caused by the different movement directions depending on the longitudinal arrangement of sensors [26]. As mentioned above, sensors 2 and 3 are located near the chest and stomach, respectively. Thus, different timings of expansion and contraction of the stomach and thorax are reflected in the signal [27].

The amplitudes of the BCG signals vary depending on their location, and the BCG waveform recorded from sensor 2 shows maximum IJ amplitude (Figure 3h). Because sensor 2 is the nearest to the heart, it can be assumed that the distance between the vibration source and the sensor affects the signal attenuation. Clean BCG waveforms are observed in the signal from sensor 1, and these are particularly less interfered by respiratory movements. In the experiment, the subject’s head was located near sensor 1; we assume that this posture feature is related to the phenomena. We assume that the location of the head affects the interference of the respiratory movement. The relative dominance of cardiac fluctuations in the area near the head is often found from our past experiences, even research approaches for embedding force transducers in pillows to monitor cardiac cycles have been reported [28].

The biggest challenge in monitoring the heart rate using BCG technology is finding features. Because it is generally known that the J peak has the largest amplitude, algorithms for estimating the heart rate by detecting the J peak have been developed. However, unlike an ECG signal having one special fiducial point, that is, QRS complex, the several waveform peaks of the BCG signal (labeled H through N) have similar size and shape. Furthermore, because the BCG signal waveform varies a lot depending on the sensor used and the position at which it was measured, it is not easy to develop a robust algorithm that considers all these characteristics. Brüser *et al.* developed and verified a new algorithm that can measure the beat-to-beat interval that corresponds to a heartbeat, unlike the conventional method for finding the location of the J peak [25]. It is difficult to expect the J peak to be dominant for an infant BCG signal processed in this study because of the nature of a premature heart. We also thought that it was more important to find the corresponding interval to obtain the correct average heartbeat rate instead of finding the exact location of the J peak. Thus, we developed a modified algorithm based on Brüser’s method.

In this study, the sensor selection process that detects valid signals from multiple sensors was considered important, unlike in previous studies. By combining the advantages of existing methods [5,29], we developed an automatic sensor selection method using an indicator that can estimate the artifact level using the auto correlation coefficient value and area rate of PSD. We assessed the coverage and mean relative error of experiments by varying the threshold value of the indicator that decides the artifact level. There is a trade-off: if the threshold value is set high, the error as well as the coverage decrease because of the increased number of dropped signals. The threshold value from our empirical experience based on the result of this study is 0.65 for HR analysis and 1.25 for BR analysis. 

The performance of our algorithm was evaluated using experimental results, and it is summarized in Table 3, Table 4, Table 5 and Table 6. Table 3 and Table 4 show the result of evaluating how precisely the algorithm can detect the peak location in terms of TP, FP, and FN. The peak was detected at 76.16% and 89.35% in contrast to the reference signal (ECG for heartbeat and chest-belt for respiration, respectively). The increased number of FNs in HR analysis is inevitable because of the nature of the algorithm, which automatically runs the entire process of checking the quality and selecting the optimum sensor. In fact, the inevitable number of FNs that occurred in the area determined to be an artifact area was around 55% (=7469/13,417) of the total number of FNs in the HR analysis. This serves as contrasting evidence that only a clear signal was analyzed using a strict standard that is underpinned by high PPV values. Apart from traditional methods for finding the location of the J peak, the detected beat-to-beat intervals that correspond to the heartbeat and respiratory cycle were evaluated by averaging the error of the preset window. The averaged relative differences and errors are summarized in Table 5 and Table 6, respectively. 

We also summarized several related studies in Table 7. The application of BCGs to infants is very rare, and it is difficult to directly compare our method with other BCG-based techniques applied to a similar group of subjects. Erkinjuntti *et al.* reported the feasibility of the static charge sensitive bed method for the monitoring of around 40 neonates [16]. However, they showed just a partial example of the simultaneous recording of signals and did not present algorithms for HR and BR analysis. Wang *et al.* investigated a piezoelectric film (PVDF) sensor system; however, their experiments only lasted 10 min on average, and their method showed only mean peak detection errors including FP and FN. Thus, we could estimate the accuracy of the estimated HR and BR signals to have mean errors of 8.24% and 4.41%, respectively [9]. For adults, BCG-derived HR and BR measurements have achieved lower errors than in our study. Other sensors such as a static charge sensor, piezoelectric sensor (PVDF and EMFi), acceleration sensor, and optical sensor have been used for detecting BCG signals when lying down in bed. A direct comparison is difficult because each method has its own advantages. However, because the load-cell sensor used in this study has the stability in terms of long-term monitoring with high accuracy as well as can simultaneously detect static/dynamic changes, we decided that it was suitable for monitoring the biological signals of infants for a long period of time when they were lying in bed. Studies for comparing the features of various sensors processed with standardized conditions in a controlled situation are needed.

We designed a load-cell-based physiological signal monitoring bed for infants and investigated whether BCGs and respiratory signals could be obtained accurately. By analyzing the data derived from 13 experiments with four infants, we verified that the proposed BCG-based technology could estimate both HR and BR information with average errors of 2.55% and 2.66%, respectively, compared with reference equipment. Although our results demonstrate that the proposed method shows acceptable performance, there are two main limitations in our study—insufficient recording time and too few measurements. Future research projects should increase the number of subjects in the experiment and extend the age distribution. The recording times should be increased to cover various occurrences of all possible types and combinations of movements during sleep and wakefulness. However, despite these limitations, the preliminary results provide a positive feasibility for future studies of BCG-based measurements in infants. We believe that the proposed device and algorithm presented here are essential steps toward substantiating the unobtrusive physiological measurement for infants. The proposed technology could be used for the continuous observation of infants, especially to detect respiratory distress and cardiac abnormalities. Also we expect extensive applications in the field of sleep research for analyzing sleep efficiency and structures of infants. 

## Figures and Tables

**Table 1:** Technical specifications of load-cell sensor.

Single Point Load-Cell	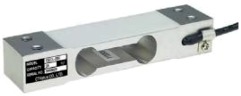
Load-cell material	Anodized Aluminum
Gage factor	2.05
Rated output	2.0 ± 0.2 mV/V
Repeatability	0.01 (% Rated output)
Creep	0.03 (% Rated output, 30 min)
Zero balance	0 ± 0.1 mV/V
Maximum excitation	15 V (DC)
Maximum capacity	6 kg
Input resistance	400 ± 20 Ω
Output resistance	350 ± 3.5 Ω

**Table 2:** Physical information about the participating subjects and experimental time.

Records	Gender	Age (Months)	Height (cm)	Weight (kg)	Experimental Time (min)	Data Length * (Sample)
A-1	Female	42	97.5	14.5	12	720,000
A-2		43	97.8	15.0	20	1,200,000
A-3		44	98.0	15.2	25	1,500,000
A-4		46	98.5	15.5	30	1,800,000
A-5		48	99.2	15.9	30	1,800,000
B-1	Male	13	78.0	13.0	10	600,000
B-2		14	78.5	13.3	20	1,200,000
B-3		19	80.1	14.8	90	5,400,000
C-1	Male	09	71.0	9.20	11.9	714,000
C-2		09	71.0	9.20	178.8	10,728,000
C-3		10	72.0	9.40	30	1,800,000
C-4		13	77.0	10.0	72	4,320,000
D-1	Male	05	67.0	9.20	33	1,980,000

*: Data were collected at a sampling rate of 1000 Hz.

**Table 3:** Performance evaluation results with respect to the beat locations in HR analysis.

Records	Time (min)	Total Beat (ECG)	Total Beat (Load-Cell)	TP	FP	FN	FN * (Inevitable)	Detection Rate	PPV
A-1	12	995	677	666	11	329	141	66.93	98.38
A-2	20	1785	1754	1748	6	37	3	97.93	99.66
A-3	25	2298	1754	1730	24	568	288	75.28	98.63
A-4	30	2868	1368	1327	41	1541	1027	46.27	97.00
A-5	30	2270	2122	2120	2	150	107	93.39	99.91
B-1	10	1114	444	435	9	679	307	39.05	97.97
B-2	20	1968	1034	1009	25	959	453	51.27	97.58
B-3	90	8651	7624	7566	58	1085	546	87.46	99.24
C-1	11.9	1153	1063	1053	10	100	18	91.33	99.06
C-2	178.8	18,481	15,399	15,244	155	3237	1402	82.48	98.99
C-3	30	3235	2884	2867	17	368	114	88.62	99.41
C-4	72	7874	5549	5516	33	2358	1101	70.05	99.41
D-1	33	3598	1603	1592	11	2006	1962	44.25	99.31
Total	562.7	56,290	43275	42,873	402	13,417	7469	76.16	99.07

*: FN (inevitable) represents the number of FN results that inevitably occur in an area determined to be an artifact.

**Table 4:** Performance evaluation results with respect to the beat locations in BR analysis.

Records	Time (min)	Total Beat (Chest Belt)	Total Beat (Load-Cell)	TP	FP	FN	FN * (Inevitable)	Detection Rate	PPV
A-1	12	Respiration was not recorded in these experiments							
A-2	20	396	379	377	2	19	0	95.20	99.47
A-3	25	509	452	445	7	64	0	87.43	98.45
A-4	30	344	262	230	32	114	0	66.86	87.79
A-5	30	504	490	472	18	32	0	93.65	96.33
B-1	10	Respiration was not recorded in these experiments							
B-2	20	284	275	248	27	36	0	87.32	90.18
B-3	90	1598	1575	1480	95	118	0	92.62	93.97
C-1	11.9	301	273	271	2	30	0	90.03	99.27
C-2	178.8	2699	2521	2409	112	290	0	89.26	95.56
C-3	30	865	789	779	10	86	0	90.06	98.73
C-4	72	1779	1580	1551	29	228	0	87.18	98.16
D-1	33	824	786	765	21	59	0	92.84	97.33
Total	540.7	10,103	9382	9027	355	1076	0	89.35	96.22

*: FN (inevitable) results did not occur in BR analysis. Because the chest belt also consists of a force transducer, the reference signal is simultaneously interfered by a motion artifact. (We could not label the respiratory peak in both reference signals and load-cell signals.)

**Table 5:** Comparison of HRs calculated from commercial device and proposed device.

Records	Time (min)	Window (Number) **	Coverage (%)	Mean HR from ECG (bpm)	Mean HR from Load-Cell (bpm)	Mean Difference * (bpm)	Mean Absolute Error * (%)
A-1	12	102	67.02	83.33	83.92	2.25	2.66
A-2	20	250	98.39	89.29	89.46	1.07	1.18
A-3	25	250	78.99	90.70	89.45	2.35	3.14
A-4	30	202	52.61	89.65	86.64	3.96	5.24
A-5	30	328	89.19	83.45	83.36	0.68	0.83
B-1	10	52	41.91	110.31	106.15	5.19	5.82
B-2	20	137	54.29	96.44	95.44	2.67	3.05
B-3	90	1026	89.71	95.17	94.66	1.47	1.69
C-1	11.9	137	92.18	96.84	97.14	1.38	1.43
C-2	178.8	1928	85.82	101.66	100.53	2.24	2.58
C-3	30	339	90.02	106.90	106.90	0.90	0.87
C-4	72	655	72.54	107.17	106.53	1.95	1.94
D-1	33	193	46.61	106.47	105.13	2.52	2.66
Average	43.28	430.69	73.79	96.72	95.79	2.20	2.55

*: Between HRs from ECG and BCG from load-cell data; **: Total number of short analysis windows that were used for HR analysis.

**Table 6:** Comparison of BRs calculated from commercial device and proposed device.

Records	Time (min)	Window (Number) **	Coverage (%)	Mean BR from Chest Belt (bpm)	Mean BR from Load- Cell (bpm)	Mean Difference * (bpm)	Mean Absolute Error * (%)
A-1	12	Respiration was not recorded in these experiments					
A-2	20	40	97.53	19.81	19.80	0.19	0.96
A-3	25	46	88.75	20.32	20.31	0.32	1.58
A-4	30	29	46.77	17.57	18.41	0.95	4.75
A-5	30	54	85.87	18.57	18.80	0.33	1.74
B-1	10	Respiration was not recorded in these experiments					
B-2 ***	20	27	78.31	19.48	20.88	1.65	7.82
B-3	90	163	87.95	19.17	19.75	0.63	3.08
C-1	11.9	22	92.94	25.23	25.22	0.06	0.24
C-2 ***	178.8	200	82.36	25.80	25.68	0.60	2.42
C-3	30	56	91.04	29.28	28.91	0.71	3.07
C-4	72	128	85.76	25.22	25.28	0.31	1.21
D-1	33	61	89.49	26.17	26.27	0.62	2.37
Average	49.15	75.09	84.25	22.42	22.66	0.58	2.66

*: Between BRs from chest belt and respiratory signal from load-cell data; **: Total number of short analysis windows that were used for BR analysis; ***: There was slight signal loss in the reference breathing signal. These sections were excluded in the analysis.

**Table 7:** Performance summary of other related works.

Record	Subjects and Detail	Sensor	Average Recording Time (min)	HR or BR	Mean Coverage (%)	Mean Difference (bpm)	Mean Absolute Error (%)	Reference
01	1822 ± 20 g of 5 premature infants (M = 1, F = 4)	PVDF	10	HR	-	-	4.41	[9]
				BR	-	-	8.24	
02	26.5 ± 0.7 year of 2 adults (M = 1, F = 1)	PVDF, EMFi	10	HR	-	1.75 ± 1.09	-	[21]
03	29 ± 5 year of 16 adults (M = 9, F = 7)	Strain gauges	26.6	HR	95.94 ± 1.28	-	1.79 ± 0.86	[22]
04	23–40 year of 13 adults (M = 13)	Air cell	5	HR	-	0.68 ± 0.77	0.98 ± 0.49	[23]
				BR	-	0.50 ± 0.63	2.85 ± 1.15	
05	49–68 year of 28 adults (M = 15, F = 13)	PVDF	540	HR	92.7	-	1.06	[24]
06	32.8 ± 13.4 year of 8 healthy adults (M = 1, F = 7)	EMFi	420	HR	84.53 ± 5.14	-	0.61 ± 0.21	[25]
	47.0 ± 13.1 year of 25 insomnia patients (M = 13, F = 12)		393	HR	68.90 ± 15.33	-	0.83 ± 0.37	
07	25.69 ± 7.13 year of 13 adults (M = 13)	PVDF	10	HR	-	0.61 ± 0.36	0.99 ± 0.56	[5]
				BR	-	0.48 ± 0.35	3.65 ± 2.89	
This study		Load-cell	43.3	HR	73.79 ± 19.37	2.20 ± 1.26	2.55 ± 1.54	
			49.2	BR	84.25 ± 13.45	0.58 ± 0.44	2.66 ± 2.11	

## Abbreviations

The following abbreviations are used in this manuscript:

ECG	Electrocardiography
BCG	Ballistocardiograph
HR	Heart rate
BR	Breathing rate
Dr	Detection rate
PPV	Positive predictive value
TP	True positive
FP	False positive
FN	False negative
